# Prescription Drug Coverage for Treatment of Low Back Pain Among US Medicaid, Medicare Advantage, and Commercial Insurers

**DOI:** 10.1001/jamanetworkopen.2018.0235

**Published:** 2018-06-22

**Authors:** Dora H. Lin, Christopher M. Jones, Wilson M. Compton, James Heyward, Jan L. Losby, Irene B. Murimi, Grant T. Baldwin, Jeromie M. Ballreich, David A. Thomas, Mark Bicket, Linda Porter, Jonothan C. Tierce, G. Caleb Alexander

**Affiliations:** 1 Center for Drug Safety and Effectiveness, Johns Hopkins Bloomberg School of Public Health, Baltimore, Maryland; 2 Department of Epidemiology, Johns Hopkins Bloomberg School of Public Health, Baltimore, Maryland; 3 Office of the Assistant Secretary for Planning and Evaluation, US Department of Health and Human Services, Washington, DC; 4 National Institute on Drug Abuse, National Institutes of Health, Bethesda, Maryland; 5 Division of Unintentional Injury Prevention, Centers for Disease Control and Prevention, Atlanta, Georgia; 6 Department of Health Policy and Management, Johns Hopkins Bloomberg School of Public Health, Baltimore, Maryland; 7 Department of Anesthesiology and Critical Care Medicine, Johns Hopkins School of Medicine, Baltimore, Maryland; 8 National Institute of Neurological Disorders and Stroke, National Institutes of Health, Bethesda, Maryland; 9 Division of General Internal Medicine, Johns Hopkins Medicine, Baltimore, Maryland

## Abstract

**Importance:**

Despite unprecedented injuries and deaths from prescription opioids, little is known regarding medication coverage policies for the treatment of chronic noncancer pain among US insurers.

**Objective:**

To assess medication coverage policies for 62 products used to treat low back pain.

**Design, Setting, and Participants:**

A cross-sectional study of health plan documents from 15 Medicaid, 15 Medicare Advantage, and 20 commercial health plans in 2017 from 16 US states representing more than half the US population and 20 interviews with more than 43 senior medical and pharmacy health plan executives from representative plans. Data analysis was conducted from April 2017 to January 2018.

**Main Outcomes and Measures:**

Formulary coverage, utilization management, and patient out-of-pocket costs.

**Results:**

Of the 62 products examined, 30 were prescription opioids and 32 were nonopioid analgesics, including 10 nonsteroidal anti-inflammatory drugs, 10 antidepressants, 6 muscle relaxants, 4 anticonvulsants, and 2 topical analgesics. Medicaid plans covered a median of 19 opioids examined (interquartile range [IQR], 12-27; median, 63%; IQR, 40%-90%) and a median of 22 nonopioids examined (IQR, 21-27; median, 69%; IQR, 66%-83%). Medicare Advantage plans covered similar proportions (median [IQR], opioids: 17 [15-22]; 57% [50%-73%]; nonopioids: 22 [22-26]; 69% [69%-81%]), while commercial plans covered more opioids (median [IQR], 23 [21-25]; 77% [70%-84%]) and nonopioids (median [IQR], 26 [24-27]; 81% [74%-85%]). Utilization management strategies were common for opioids in Medicaid plans (median [IQR], 15 [11-20] opioids; 91% [74%-97%]), Medicare Advantage plans (median [IQR], 15 [9-18] opioids; 100% [100%-100%]), and commercial plans (median [IQR], 16 [11-20] opioids; 74% [53%-94%]), generally relying on 30-day quantity limits rather than prior authorization. Step therapy was especially uncommon. Many of the nonopioids examined also were subject to utilization management, especially quantity limits (24%-32% of products across payers) and prior authorization (median [IQR], commercial plans: 2 [0-3] nonopioids; 9% [0%-11%]; Medicare Advantage plans: 4 [3-5] nonopioids; 19% [10%-23%]; Medicaid plans: 6 [1-13] nonopioids; 38% [2%-52%]). Among commercial plans, the median plan placed 18 opioids (74%) and 20 nonopioids (81%) in tier 1, which was associated with a median out-of-pocket cost of $10 (IQR, $9-$10) per 30-day supply. Key informant interviews revealed an emphasis on increasing opioid utilization management and identifying high-risk prescribers and patients, rather than promoting comprehensive strategies to improve treatment of chronic pain or better integrating pharmacologic and nonpharmacologic alternatives to opioids.

**Conclusions and Relevance:**

Given the effect of coverage policies on drug utilization and health outcomes, these findings provide an important opportunity to evaluate how formulary placement, utilization management, copayments, and integration of nonpharmacologic treatments can be optimized to improve pain care while reducing opioid-related injuries and deaths.

## Key Points

**Question:**

Among US insurers, what are the coverage policies for pharmacologic treatments for low back pain?

**Findings:**

In this cross-sectional study of 62 products used to treat low back pain examined across 50 Medicaid, Medicare Advantage, and commercial insurance plans, utilization management strategies were common for nonopioids and opioids alike. Key informant interviews with plan executives underscored the frequent absence of comprehensive strategies to improve chronic pain treatment and to better integrate pharmacologic and nonpharmacologic opioid alternatives.

**Meaning:**

Our findings underscore important opportunities among insurers to redesign coverage policies to improve pain management and reduce opioid-related injuries and deaths.

## Teaser

This study compares medication coverage policies and utilization management strategies of US Medicaid, Medicare Advantage, and commercial insurers for 62 products used to treat low back pain, including prescription opioids and nonopioid analgesics.

## Introduction

Between 1999 and 2010, opioid-related overdose deaths rose markedly in parallel with the increased prescribing of opioids in the United States,^1^ reaching a total of 42 249 deaths in 2016.^2^ An estimated 25 million Americans experience pain every day, often significantly interfering with daily activities.^3^ Many experts^4^ as well as recent projections^5^ suggest that the rates of injuries and deaths from opioids will continue to increase in the near term, underscoring the urgency for comprehensive, coordinated interventions focused on improving pain care and reducing opioid-related harms.

The Centers for Disease Control and Prevention guideline for prescribing opioids for chronic pain^6^ and clinical practice guidelines for low back pain^7,8^ set forth evidence-based recommendations and promote use of nonopioid therapies as first-line treatment for chronic pain. The National Pain Strategy calls for a multipronged approach to provide integrated care to treat chronic pain.^9^ It also highlights the need to improve coverage and reimbursement policies, because these policies play an important role in shaping drug utilization.^10,11^ Despite this, little is known regarding how public and private payers have designed coverage policies for opioids and other treatments for chronic noncancer pain.^9^ One recent study examined utilization management over the past decade and found that Medicare Part D formularies increasingly used quantity limits and, to a lesser degree, prior authorization to restrict opioid prescribing.^12^ Others have highlighted coverage gaps for opioid use disorder treatment^13^ and its potential unintended consequences.^14^ However, the persistent information gap regarding coverage of chronic noncancer pain treatments constrains the ability of decision makers to develop informed policies.

We evaluated the 2017 coverage policies of a diverse group of Medicaid, Medicare Advantage, and commercial health plans. We focused on 62 pharmacologic treatments for low back pain because it is one of the most common causes of chronic pain^15^ and one of the conditions for which prescription opioids have been commonly overused despite an unfavorable risk-benefit profile.^16,17^ We supplemented our data analysis with 20 key informant interviews with medical and pharmacy directors. In addition to examining any systematic differences in coverage of prescription opioids compared with nonopioids, we examined the prevalence of utilization management and how this varied across plans and payers, as well as products’ cost sharing. We hypothesized that utilization management strategies such as prior authorization and quantity limits would often be used for nonopioid as well as opioid products and that plans would report relatively little integration of nonpharmacologic and pharmacologic approaches to the management of chronic noncancer pain.

## Methods

We conducted a cross-sectional pilot study from June 16, 2016, through September 14, 2016; the upscaled study was conducted from April 14, 2017, through January 31, 2018. In our pilot study, we evaluated national coverage documents from a large commercial insurance plan, Medicaid program, and pharmacy benefits manager, refining our product selection list, gaining familiarity with the publicly available variables in plan documents, and designing data extraction forms and quality controls. In the current study, we conducted a mixed-methods analysis of coverage policy, whereby quantitative analyses of coverage documents were used to inform key informant interviews, and interviews were used to assist in interpreting results from our quantitative analyses. Our study fulfills the Strengthening the Reporting of Observational Studies in Epidemiology (STROBE) reporting guideline and Standards for Reporting Qualitative Research (SRQR) reporting guideline. We obtained informed consent from all informants prior to interviewing them. This study was exempted from a Johns Hopkins Bloomberg School of Public Health institutional review board review, given that no personal or private information was collected from informants. Participants provided verbal consent to use information from the interviews, including direct quotations, without attribution.

### Product and Plan Selection

We used an iterative process (eAppendix in the Supplement) to select 62 pharmaceutical products of interest commonly used for the treatment of low back pain (eTable 1 in the Supplement). We selected 50 plans, which included 15 Medicaid programs, 15 Medicare Advantage plans, and 20 commercial plans of insurers and plan types that cover the majority (72%) of Americans.^18,19^ We used a representative sampling strategy to simultaneously achieve several goals, including maximizing geographic diversity and assessing states with diverse and differently sized populations (eAppendix in the Supplement). We included 16 states that in aggregate represent more than half of the US population, as well as several or that have been disproportionately affected by the opioid overdose epidemic, such as Ohio and West Virginia (Figure). eTable 2 in the Supplement lists our final selection of plans.

**Figure.**
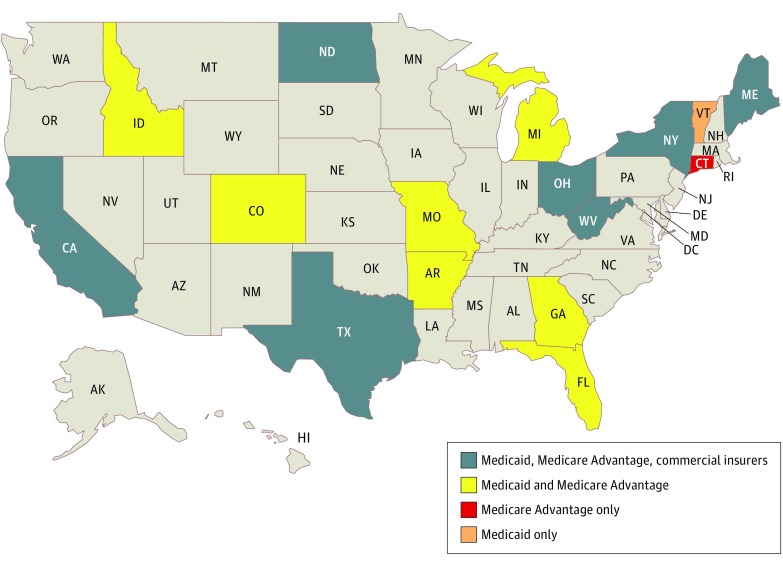
States for Which Coverage Policies Were Analyzed

### Key Informant Selection

We conducted 20 key informant interviews with more than 43 informants within many of the identified payers, including 6 interviews representing Medicaid plans, 2 Medicare Advantage or Part D plans, 9 commercial plans, and 3 trade organizations (eg, Blue Cross/Blue Shield Association of America). We focused on senior executives responsible for the design, implementation, and evaluation of medical and pharmacy policies within the payer, such as chief medical officers, chief pharmacy officers, and vice presidents of clinical operations.

### Health Plan Data Extraction

We identified publicly available, health plan–specific coverage documents from the internet, including the 2017 plan formulary, summary of benefits and coverage, and evidence of coverage. Three commercial plans examined did not provide a publicly available plan-specific formulary; as such, we extracted data from a national or regional formulary for these plans instead. Each policy document was abstracted by a single reviewer. A second reviewer abstracted 20% of the data, with resulting interrater agreement exceeding 95% and discrepancies resolved by study team consensus. Outcome measures included health plan characteristics and chronic pain policies regarding pharmaceutical coverage and utilization management such as prior authorization, quantity limits, and cost sharing. We defined a medication as not covered if it was not listed on a formulary.

### Interview Data Collection

Individuals were contacted by email and interviewed by telephone using a semistructured script that was developed and iteratively piloted and pretested to maximize the value of the qualitative information received. An interviewer and a research analyst were present for each call, and key comments were transcribed verbatim. The interviews covered 5 key domains: (1) plan responses to the opioid epidemic, (2) coordination between pharmacologic and nonpharmacologic treatments, and the development of (3) innovative strategies, (4) requirements, or (5) technologies to improve the care of patients with chronic noncancer pain.

### Statistical Analysis

We cleaned the extracted data and used visual inspection to examine data distributions. We then used descriptive statistics to characterize coverage policies and utilization management requirements across insurers and therapeutic classes. To analyze our key informant interviews, we used a grounded theory approach.^20^ We organized each interviewee’s comments around our 5 broad study domains and identified illustrative quotes to support the insights derived. Next, we generated a new study document that, for a given domain, listed the diversity of feedback that we received regarding the topic. Finally, we iteratively synthesized this feedback in narrative form.

## Results

Of the 62 products examined, 30 were prescription opioids. The remaining 32 were nonopioid analgesics, including 10 nonsteroidal anti-inflammatory drugs (NSAIDs), 10 antidepressants, 6 muscle relaxants, 4 anticonvulsants, and 2 topical analgesics.

### Formulary Coverage

The overall proportions of opioids and nonopioids covered by a given payer were generally similar, with commercial plans covering the most products. However, 2 plans covered significantly more opioids than nonopioids; conversely, 7 plans covered significantly more nonopioids than opioids. Table 1 shows coverage of the products we examined across the plans. For example, of the 30 prescription opioids examined, the Medicaid plans covered a median of 19 (interquartile range [IQR], 12-27; median, 63%; IQR, 40%-90%) of these products. A similar proportion was covered by Medicare Advantage plans (median [IQR], 17 [15-22]; 57% [50%-73%]), whereas more than three-fourths of products (median [IQR], 23 [21-25]; 77% [70%-84%]) were covered by the commercial plans. eTable 3 in the Supplement shows the coverage for each examined opioid product across individual plans.

**Table 1.** Covered Products for Treatment of Pain Among 50 Health Plans

Drug Class	Median (IQR)							
	Medicaid Plans (n = 15)		Medicare Advantage Plans (n = 15)		Commercial Plans (n = 20)		All Plans (N = 50)	
	No.	%	No.	%	No.	%	No.	%
Opioids (n = 30)	19 (12-27)	63 (40-90)	17 (15-22)	57 (50-73)	23 (21-25)	77 (70-84)	22 (15-25)	72 (50-83)
Immediate-release (n = 17)	11 (9-16)	65 (50-94)	12 (11-14)	71 (65-82)	15 (13-16)	85 (76-94)	14 (10-16)	82 (60-94)
Extended-release (n = 13)	9 (4-12)	69 (31-88)	4 (3-8)	31 (23-62)	9 (7-11)	65 (54-81)	8 (4-11)	62 (31-83)
Nonopioids (n = 32)	22 (21-27)	69 (66-83)	22 (22-26)	69 (69-81)	26 (24-27)	81 (74-85)	24 (21-27)	75 (66-84)
NSAIDs (n = 10)	8 (8-9)	80 (75-90)	9 (9-10)	90 (90-100)	8 (6-10)	80 (63-98)	9 (7-10)	90 (73-100)
Antidepressants (n = 10)	7 (6-8)	70 (60-75)	8 (8-9)	80 (80-90)	8 (7-9)	80 (73-90)	8 (7-9)	80 (70-90)
Anticonvulsants (n = 4)	2 (2-4)	50 (38-100)	2 (2-4)	50 (50-100)	3 (2-4)	75 (50-100)	3 (2-4)	63 (50-100)
Topical analgesics (n = 2)	1 (1-2)	50 (50-100)	2 (1-2)	100 (50-100)	2 (1-2)	100 (50-100)	2 (1-2)	100 (50-100)
Muscle relaxants (n = 6)	5 (3-6)	83 (50-100)	1 (1-3)	17 (17-42)	5 (4-5)	83 (67-83)	4 (3-5)	67 (50-83)

Abbreviations: IQR, interquartile range; NSAIDs, nonsteroidal anti-inflammatory drugs.

Trends were similar for the nonopioids examined, with a greater proportion of nonopioids covered by commercial plans (median [IQR], 26 [24-27]; 81% [74%-85%]) than Medicaid plans (median [IQR], 22 [21-27]; 69% [66%-83%]) or Medicare Advantage plans (median [IQR], 22 [22-26]; 69% [69%-81%]).

Medicare Advantage and commercial plans had greater coverage for immediate-release opioids (median [IQR], 12 [11-14]; 71% [65%-82%] and 15 [13-16]; 85% [76%-94%], respectively) than extended-release opioids (median [IQR], 4 [3-8]; 31% [23%-62%] and 9 [7-11]; 65% [54%-81%], respectively), whereas Medicaid had approximately the same coverage for both types (median [IQR], immediate-release opioids: 11 [9-16]; 65% [50%-94%] and extended-release opioids: 9 [4-12]; 69% [31%-88%]). Among all plans, a larger proportion of the examined NSAIDs (median [IQR], 9 [7-10]; 90% [73%-100%]) and antidepressants (median [IQR], 8 [7-9]; 80% [70%-90%]) were covered than anticonvulsants or muscle relaxants.

Our 20 key informant interviews provided insight into the context for the current formulary coverage of pain medications; plans were universally active in modifying coverage for pain treatments to decrease the volume of opioids prescribed (Table 2). For example, plans have widely begun to implement elements of the 2016 Centers for Disease Control and Prevention guidelines for prescribing opioids for chronic pain, most commonly by limiting opioid prescriptions through the implementation of morphine milligram equivalent limits as well as other quantity or duration limits. Other plans reported having taken, or planning to imminently take, additional measures, some in coordination with other insurers and statewide entities also working to address the epidemic.

**Table 2.** Key Themes and Illustrative Quotes From 20 Interviews With Medicaid, Medicare Advantage, and Commercial Payers With Respect to Opioid Epidemic and Prescription Drug Coverage Policy

Key Themes	Illustrative Quotes
Insurers universally modifying coverage policies for pain treatments	“We have this holistic approach that we have deployed, it has impacted how we cover opioid prescriptions, and how we work with prescribers around those prescriptions, and also access to treatment for those who are addicted to opioids, such as medication assisted treatment.” [MD-8]
	“We decided to focus on 2 groups of overutilizers: the poorly coordinated where you have 4 different prescribers and 4 pharmacies, and the uneducated doctors using huge doses, but do not realize they can get addicted.” [MD-10]
	“One of the things we committed to in our work with the National Governors’ Association was the creation of an expert work group to work with them to develop best practices [for combating the opioid epidemic], for [our plan’s] system-wide adoption in 2016. By summer we had created 4 best practices by pulling policies from specific plans and consensus building.” [MD-12]
	“Every plan in the country is aware that this is an issue… it’s just that they may not have the time, energy or in-house expertise to address it. Some plans are awfully further ahead than others. There’s a lot of innovation and a lot of approaches.” [MD-6]
Most plans targeting high-risk patients and prescribers	“We identified patients using high doses of opioids, multiple pharmacies, multiple prescribers and we restricted them to specific pharmacies, which cut down on their… prescribing.” [MD-6]
	“They [pharmacy benefit managers] are trying to keep track, especially of those patients that tend to be frequent fliers in emergency rooms or going to multiple physicians, and are trying to make sure, with the aid of case managers, that they are connected with pain management specialists, and if they do not have access because live in a remote area or something, we try to have providers make sure they are using pain management contracts with them, identifying the pharmacy provider that they are going to use.” [MD-2]
	“[We are] also targeting providers with outlier members, taking into account the diagnosis to eliminate false positives (e.g. cancer pain). Once contacted the prescriber has chance to contact a nurse… or plan pharmacist… for more information. This is one of our most responded-to campaigns. Prescribers feel as if there are repercussions if they don’t respond.” [PD-2]
	“We figured out who the high prescribers were and gave them a report card. The strategy was to identify them, notify them, give them information on best practices, and if they did not change… eliminate them from the network.” [MD-6]
	“We are very concerned that some providers in the United States are over prescribing, which dovetails with our efforts to notify certain providers that are super prescribers, through letter writing and other, more aggressive methods to deter over prescribing.” [MD-8]
Focus on reducing opioid overuse rather than increasing use of nonopioids	“The established benefits for nonopioid treatments for long-term pain are just not fair… many die at the hands of unintended opioid use and I really hope the guidelines are taken to heart by providers.” [PD-3]
	“Whether we have encouraged plans to improve access, loosen restrictions to nonopioids in conjunction with improving the safety of opioids—I am not aware of policies in that regard.” [PD-6]
	“In response to the opioid epidemic specifically, [we] have added a number of clinical edits. Quantity limits for opioids have been the primary utilization management strategy, as well as limits on the number of prescriptions for certain drugs over time.” [PD-1]
	“In 2016, we put a limit of 300 morphine milligram equivalent per day on all opiates together… in October 2017 we are taking that down to 250 morphine milligram equivalent, and we have plans to slowly take that down.” [MD-5]
	“We don’t have provider networks that support [expansion of nonpharmacological benefits], and we do not have ready and willing patients.… For a lot of these things, they either have to pay out of pocket, or else they are stuck with a very limited set of free services.” [PD-5]
Generally poor linkage between pharmacy and medical policy	“That is… something we really grapple with. As a payer we are loath to get in the way of the therapeutic alliance of the patient and the doctor, we don’t intervene unless there’s an access issue. We don’t tell doctors how to manage the condition…. We can’t dictate how pain gets treated—we can only remove barriers to access.” [MD-8]
	“Our coverage policies for pharmacologic and nonpharmacologic used to be coordinated, but we lost a bit of coordination when we moved to delegated formularies as of January 1, 2017. CVS and Express Scripts are really leading the efforts for making frameworks for dealing with opioid policies.” [PD-3]
	“We are thinking about it now. It is… clear that operating within silos where each goes on their merry way just simply won’t work, and we want providers to think through alternative medicine and nonpharmacologic pain management. There’s a lack of knowledge and transparency with respect to what’s covered… we want to make sure providers are aware of patient’s nonpharmacy benefits for controlling pain.” [PD-2]
	“We are not at that level yet, [and do not have] coordination between drug and non-drug benefits. In a perfect situation, I would like to see certain limitations placed [on opioid therapy], maybe, look at diagnostic categories, certain diagnosis might require more, certain diagnosis might require less, look at quantity limits from that point of view, and coordinate that with what are the nonmedication activities that might enhance care for the condition, instead of filling for more opioids, more opioids, more opioids.” [MD-2]
	“They are not coordinated in any particular way at this point… the pharmacy people manage the pharmacy and the medical people manage the devices... I could see them talking and coordinating, but… I do not see a plan which is so huge and has so many moving pieces doing it themselves. We would need a vendor to guide us. It is just too complicated and we have too many benefit designs.” [MD-6]
Some payers adopting innovative strategies, others desire to do so	”[One ongoing program is] to encourage emergency department docs to start prescribing medication assisted treatment in the emergency department…. The research shows that if you engage a member at a vulnerable moment such as in the ER, when they are experiencing an overdose, you have the opportunity to initiate MAT along with wraparound therapy services.” [MD-8]
	“We currently have an academic detailing pilot in three counties to do outreach to the high [volume] prescribers… and we are sitting down with those prescribers to talk about opioid safety, and non-opioid interventions, and of course naloxone, and access to buprenorphine and methadone, if it exists.” [PD-5]
	“We are using mindfulness to combat chronic pain for members, and this approach is available to members through large employers… we [also] have 14 000 employees that have gone through mindfulness training” [MD-8]
	“We just partnered with Walgreens to put in ‘drug takeback boxes’ across the country. And, from a more proactive stand, we are trying educate people about the potential for fraud or diversion when they leave these things [opioids] in their medicine cabinet.” [MD-12]
	“We have a program on shared decision-making; one area was back pain. We had [a vendor] with a number of ways to do this: a module, coach, or app, such as modules on pain management. This helped put people into lower cost options.” [MD-6]
	“It is now required for all [Medicare Advantage] plan sponsors to use a point-of-sale safety edit that fires when a claim for a beneficiary would cause the morphine milligram equivalent dose to exceed the limit that the plans’ pharmacy and therapeutics committee set. We set expected limits in our call letter, depending on if it’s a soft or hard edit. Sponsors can also include a prescriber and pharmacy count in the criteria.” [PD-6]

Abbreviations: ER, emergency room; MAT, medication-assisted treatment; MD, medical director; PD, pharmacy director.

### Utilization Management for Opioids

Utilization management strategies were common for opioids, with at least 1 form of utilization management for a median of 15 opioids (IQR, 11-20; median, 91%; IQR, 74%-97%) in Medicaid plans, 15 (IQR, 9-18; median, 100% [IQR, 100%-100%]) in Medicare Advantage plans, and 16 (IQR, 11-20; median, 74% [IQR, 53%-94%]) in commercial plans, generally relying on 30-day quantity limits rather than prior authorization or step therapy (Table 3). For example, among Medicaid plans, a median of 11 covered opioids (IQR, 10-15; median, 69%; IQR, 45%-89%) had quantity limits, 8 (IQR, 1-15; median, 42%; IQR, 8%-69%) required prior authorization, and 1 (IQR, 0-7; median, 9%; IQR, 0%-29%) required step therapy. In all cases where quantity limits were observed, the limits provided for the daily use of each product during a 30-, 60-, or 90-day period and failed to distinguish between the first prescription and subsequent prescriptions, as opposed to 7- or 10-day prescribing limits for first fills that are increasingly common in state laws^21,22^ and payer policies.^23^ All covered opioids within Medicare Advantage plans had quantity limits but negligible use of either step therapy or prior authorization. Commercial plans also relied on quantity limits for 16 opioids (IQR, 11-20; median, 70%; IQR, 53%-94%), with infrequent use of prior authorization (median [IQR], 4 [1-5]; 15% [4%-28%]) or step therapy (median [IQR], 1 [0-2]; 4% [0%-11%]) for opioids. The restrictiveness of prior authorization requirements varied from plan to plan, with some requiring a medical rationale for the continuation of opioid therapy beyond 2 prescriptions, and others requiring only a diagnosis of chronic pain for authorization of successive prescriptions.

**Table 3.** Covered Opioid and Nonopioid Medications for Treatment of Pain With Utilization Management Among 50 Health Plans

Plans (N = 50)	Median (IQR)							
	Prior Authorization		Step Therapy		Quantity Limits		≥1 Utilization Management Tool	
	No. of Products	%^a^	No. of Products	%^a^	No. of Products	%^a^	No. of Products	%^a^
Medicaid plans (n = 15)								
Opioids	8 (1-15)	42 (8-69)	1 (0-7)	9 (0-29)	11 (10-15)	69 (45-89)	15 (11-20)	91 (74-97)
Immediate-release	1 (0-8)	13 (0-60)	0 (0-1)	0 (0-15)	8 (5-11)	84 (56-99)	9 (8-12)	88 (65-100)
Extended-release	5 (1-9)	60 (23-73)	1 (0-3)	8 (0-33)	3 (2-6)	65 (25-95)	7 (4-11)	92 (91-100)
Nonopioids	6 (1-13)	38 (2-52)	1 (0-8)	4 (0-33)	5 (2-8)	24 (10-38)	11 (7-17)	52 (34-65)
NSAIDs	1 (0-4)	13 (0-44)	0 (0-2)	0 (0-16)	1 (0-2)	11 (0-17)	2 (1-4)	25 (11-44)
Antidepressants	2 (0-5)	24 (0-71)	0 (0-3)	0 (0-31)	2 (1-4)	21 (11-43)	5 (1-6)	58 (20-85)
Anticonvulsants	2 (0-2)	50 (0-73)	0 (0-2)	0 (0-63)	1 (0-1)	25 (0-50)	2 (1-3)	71 (50-100)
Topical analgesics	1 (0-1)	25 (0-100)	0 (0-0)	0 (0-0)	1 (0-1)	50 (0-100)	1 (1-2)	100 (25-100)
Muscle relaxants	2 (0-3)	37 (0-50)	0 (0-3)	0 (0-43)	1 (0-3)	10 (0-53)	3 (1-3)	50 (27-75)
Medicare Advantage plans (n = 15)								
Opioids	0 (0-1)	0 (0-2)	0 (0-0)	0 (0-0)	15 (9-18)	100 (100-100)	15 (9-18)	100 (100-100)
Immediate-release	0 (0-0)	0 (0-0)	0 (0-0)	0 (0-0)	12 (7-14)	100 (100-100)	12 (7-14)	100 (100-100)
Extended-release	0 (0-0)	0 (0-0)	0 (0-0)	0 (0-0)	3 (2-6)	100 (100-100)	3 (2-6)	100 (100-100)
Nonopioids	4 (3-5)	19 (10-23)	1 (0-2)	4 (0-6)	7 (5-8)	32 (23-36)	10 (7-11)	45 (27-46)
NSAIDs	0 (0-0)	0 (0-0)	0 (0-0)	0 (0-0)	1 (1-2)	11 (11-20)	1 (1-2)	11 (11-21)
Antidepressants	2 (0-2)	22 (0-25)	0 (0-2)	0 (0-16)	2 (2-3)	29 (25-38)	4 (3-4)	50 (28-57)
Anticonvulsants	0 (0-1)	0 (0-13)	0 (0-0)	0 (0-0)	1 (1-2)	50 (50-100)	1 (1-2)	50 (50-100)
Topical analgesics	1 (1-2)	100 (75-100)	0 (0-0)	0 (0-0)	1 (1-1)	50 (25-100)	1 (1-2)	100 (75-100)
Muscle relaxants	1 (0-1)	50 (0-100)	0 (0-0)	0 (0-0)	0 (0-1)	0 (0-33)	1 (1-1)	50 (33-100)
Commercial plans (n = 20)								
Opioids	4 (1-5)	15 (4-28)	1 (0-2)	4 (0-11)	16 (11-20)	70 (53-94)	16 (11-20)	74 (53-94)
Immediate-release	0 (0-0)	0 (0-0)	0 (0-0)	0 (0-0)	6 (3-14)	52 (17-92)	7 (3-14)	60 (17-92)
Extended-release	4 (1-5)	33 (11-71)	1 (0-2)	10 (0-25)	8 (7-9)	100 (89-100)	8 (7-9)	100 (89-100)
Nonopioids	2 (0-3)	9 (0-11)	2 (1-3)	8 (4-12)	7 (5-8)	28 (20-35)	9 (7-10)	35 (28-38)
NSAIDs	0 (0-0)	0 (0-0)	0 (0-1)	0 (0-10)	1 (1-1)	14 (10-20)	1 (1-2)	15 (10-20)
Antidepressants	0 (0-1)	0 (0-11)	1 (0-1)	11 (0-14)	3 (2-4)	38 (30-50)	4 (3-4)	44 (32-50)
Anticonvulsants	1 (0-2)	25 (0-67)	0 (0-1)	0 (0-50)	2 (0-2)	50 (0-67)	2 (1-3)	67 (50-75)
Topical analgesics	0 (0-0)	0 (0-0)	0 (0-0)	0 (0-0)	1 (0-1)	75 (13-100)	1 (0-2)	50 (0-100)
Muscle relaxants	0 (0-0)	0 (0-0)	0 (0-0)	0 (0-0)	0 (0-0)	0 (0-0)	1 (0-1)	20 (0-27)

Abbreviations: IQR, interquartile range; NSAIDs, nonsteroidal anti-inflammatory drugs.

^a^ Percentages indicate median (IQR) percentage of products with utilization management of total covered products.

### Utilization Management for Nonopioids

Approximately 1 in 4 nonopioids was restricted through quantity limits across the examined payers, with modestly lower rates in Medicaid plans (median [IQR], 5 [2-8] nonopioids; 24% [10%-38%]) than Medicare Advantage plans (median [IQR], 7 [5-8] nonopioids; 32% [23%-36%]) and commercial plans (median [IQR], 7 [5-8] nonopioids; 28% [20%-35%]). Prior authorization was especially common in Medicaid, with a median of 6 covered nonopioids (IQR, 1-13; median, 38%; IQR, 2%-52%) restricted, as compared with a median of 4 nonopioids (IQR, 3-5; median, 19%; IQR, 10%-23%) in Medicare Advantage plans and a median of 2 (IQR, 0-3; median, 9%; IQR, 0%-11%) in commercial plans. For example, among the 11 Medicaid plans covering celecoxib, which is an NSAID, 7 (64%) had prior authorization requirements, although within-class alternatives like ibuprofen and naproxen were covered without restriction in the same plans. Prior authorization was also common for serotonin and norepinephrine reuptake inhibitor–class antidepressants, among which the prevalence of prior authorization ranged from 31% (duloxetine) to 86% (levomilnacipran) among Medicaid plans. Across all payers, the prevalence of prior authorization varied by drug class, with more restrictions on muscle relaxants (25% of covered products) than antidepressants (17.4%) or NSAIDs (6.7%). As with opioid products, the use of step therapy was uncommon. Utilization management appeared more frequently or at similar rates for opioids than nonopioids across all plans and all utilization management methods, with the exception of prior authorization for Medicare Advantage plans, which was more frequent for nonopioids (median [IQR], 4 [3-5]; 19% [10%-23%]) than opioids (median [IQR], 0 [0-1]; 0% [0%-2%]).

Payers also discussed the use of nonpharmacologic therapies. Many informants identified a need for greater coordination of nonpharmacologic and pharmacologic benefits. Pharmacy policies rarely aligned with corresponding medical policies for pain treatment, in part owing to separation in the design and administration of these 2 types of benefits. Only 1 plan we interviewed had fully integrated nonpharmacological therapies into its step therapy requirements for opioid initiation.

### Coverage and Utilization Management for Specific Opioid Products

Table 4 presents the coverage and use of utilization management for specific opioid products by insurer type. Across all plan types, methadone (49 [98%]) and fentanyl (47 [94%]) were the most common extended-release/long-acting opioids covered for pain. The least frequent extended-release/long-acting opioids covered across all plans were transdermal buprenorphine (19 [38%]) and oxycodone with naltrexone (0).

**Table 4.** Coverage and Utilization Management of Extended-Release/Long-Acting Opioids for Pain Among 50 Health Plans

Covered Products	Plans, No. (%)^a^			
	Medicaid (n = 15)	Medicare Advantage (n = 15)	Commercial (n = 20)	All (N = 50)
Coverage of products				
Methadone	14 (93)	15 (100)	20 (100)	49 (98)
Fentanyl	14 (93)	15 (100)	18 (90)	47 (94)
Morphine with naltrexone	8 (53)	5 (33)	14 (70)	27 (54)
Hydrocodone bitartrate	8 (53)	7 (47)	12 (60)	27 (54)
Oxycodone hydrochloride	12 (80)	2 (13)	9 (45)	23 (46)
Buprenorphine buccal film	7 (47)	4 (27)	11 (55)	22 (44)
Buprenorphine transdermal system	7 (47)	4 (27)	8 (40)	19 (38)
Oxycodone with naltrexone^b^	0	0	0	0
Prior authorization				
Methadone	7 (50)	0	6 (30)	13 (27)
Fentanyl	5 (36)	0	6 (33)	11 (23)
Morphine with naltrexone	4 (50)	0	7 (58)	11 (41)
Hydrocodone bitartrate	6 (86)	0	3 (38)	9 (47)
Oxycodone hydrochloride	9 (75)	0	5 (56)	14 (61)
Buprenorphine buccal film	7 (100)	2 (50)	9 (82)	18 (82)
Buprenorphine transdermal system	2 (25)	0	8 (57)	10 (37)
Oxycodone with naltrexone^b^	NC	NC	NC	NC
Step therapy				
Methadone	3 (21)	0	0	3 (6)
Fentanyl	1 (7)	0	2 (11)	3 (6)
Morphine with naltrexone	0	0	7 (58)	7 (26)
Hydrocodone bitartrate	2 (29)	0	1 (13)	3 (16)
Oxycodone hydrochloride	5 (42)	0	3 (33)	8 (35)
Buprenorphine buccal film	4 (57)	0	2 (18)	6 (27)
Buprenorphine transdermal system	1 (13)	0	2 (14)	3 (11)
Oxycodone with naltrexone^b^	NC	NC	NC	NC
Quantity limits				
Methadone	6 (43)	13 (87)	14 (70)	33 (67)
Fentanyl	11 (79)	13 (87)	17 (94)	41 (87)
Morphine with naltrexone	1 (13)	5 (71)	11 (92)	17 (63)
Hydrocodone bitartrate	1 (13)	2 (50)	8 (100)	11 (55)
Oxycodone hydrochloride	6 (50)	0	9 (100)	15 (65)
Buprenorphine buccal film	2 (29)	2 (50)	11 (100)	15 (68)
Buprenorphine transdermal system	4 (50)	3 (60)	14 (100)	21 (78)
Oxycodone with naltrexone^b^	NC	NC	NC	NC

Abbreviation: NC, not covered.

^a^ Percentage denominators for covered products are the total number of plans; percentage denominators for utilization management are number of plans covering product.

^b^ Removed from market during study period.

Of the 15 Medicaid plans we examined, 14 (93%) covered methadone for pain, and among plans covering the product, 7 (50%) used prior authorization, 6 (43%) used quantity limits, and 3 (21%) used step therapy. Medicare Advantage plans most commonly used quantity limits for extended-release/long-acting opioids and never used step therapy for opioids. Commercial plans most often used quantity limits, followed by prior authorization and step therapy.

### Product Tiering and Out-of-Pocket Costs

Ten of the 15 Medicaid plans examined required a copayment for covered products, which generally was $0 to $3 and never more than $8 per prescription, and this did not differ between opioids and nonopioids. Cost sharing for the Medicare Advantage and commercial plans examined is shown in eTable 4 in the Supplement. The median (IQR) copayment per 30-day prescription in the Medicare Advantage plans examined was $4 ($2-$10) for tier 1 drugs, $17 ($11-$20) for tier 2 drugs, $47 ($45-$47) for tier 3 drugs, $100 ($95-$100) for tier 4 drugs, and coinsurance of 31% (28%-33%) for tier 5 drugs. Among Medicare Advantage plans, 5 covered opioids (29%) were in tier 2, 6 (53%) were in tier 3, and 3 (20%) were in tier 4. For nonopioids in Medicare Advantage plans, 3 (14%) were in tier 1, 9 (41%) in tier 2, and 3 (15%) in tier 3. Among commercial plans, the median plan placed 18 opioids (74%) and 20 non-opioids (81%) in tier 1. The median copayment per 30-day prescription in tier 1 opioids in the commercial plans was $10 (IQR, $9-$10).

### Innovative Strategies

In addition to plan efforts regarding coverage, utilization management, tiering, and costs of opioids and nonopioids, the key informant interviews identified important contextual information regarding decision making as well as emerging innovative strategies to address the opioid epidemic. Plan executives contextualized formulary management within a broader landscape of efforts aimed at reducing opioid prescribing, including analysis of prescribing data to identify high-volume prescribers and patients. To address outlier prescribers, interventions included written warnings, increased training and education, and in rare cases removal from the plan’s network. Some plans deployed academic detailers to the offices of high prescribers to offer one-on-one counseling about opioid risks, treatment alternatives, and buprenorphine prescribing, while others offered online modules and seminars.

Patient-level efforts included case management, pain contracts, restricted recipient programs that lock in high-risk patients to 1 prescriber and 1 pharmacy (lock-in programs), and education about behavioral and mental health resources. Some innovative plans noted prospectively identifying at-risk patients and liaising them with case managers. Others used real-time administrative claims and prescription drug monitoring data to identify and track individuals filling prescription opioids from multiple prescribers and pharmacies. The interviews reinforced the concept that insurers have largely focused on efforts to constrain opioids rather than promote comprehensive strategies to improve treatment of chronic pain or increase access to or better integrate pharmacologic and nonpharmacologic alternatives to opioids.

## Discussion

To examine how public and commercial payers have responded to the US opioid crisis, we examined contemporary coverage policies for 62 pharmacologic treatments used for the treatment of low back pain, one of the most common causes of chronic pain for which prescription opioids have been overused. While utilization management strategies were common for opioids, many of the nonopioids examined were also subject to utilization management, especially quantity limits and prior authorization. Step therapy was rarely used. Most opioids were placed on low formulary tiers and associated with copayments of $10 to $15 per prescription. Key informant interviews with plan executives underscored that plans were universally active in modifying coverage policies, although with a primary emphasis on increasing opioid utilization management and focusing on high-risk prescribers and patients rather than promoting comprehensive strategies to improve the treatment of chronic pain or increasing access to or better integrating pharmacologic and nonpharmacologic alternatives to opioids. These findings are important because of how commonly opioids are prescribed, harms that have accrued, and the important role that public and commercial insurers can play in improving the safe use of these products, a role validated by payers in our interviews.

There is a wealth of evidence regarding the association of prescription drug coverage with drug utilization and health outcomes and increasing interest in this as it applies specifically to the coverage of prescription opioids and other treatments for chronic noncancer pain.^24,25^ Despite this, our interviewees tended to emphasize utilization management and other strategies to decrease opioid overuse rather than broader initiatives to comprehensively improve the quality of care for individuals with chronic noncancer pain. This is noteworthy because improving the provision of high-quality, evidence-based pain care represents a critical opportunity to reverse the momentum of the opioid epidemic.^26^

Because of the limited evidence that long-term opioid use improves pain, function, and quality of life, nonpharmacologic therapy and nonopioid analgesics are preferred as first-line treatment for chronic pain; coverage policies should reflect this and quality improvement efforts should incentivize this.^6,15,27^ Requiring patients and health care professionals to navigate burdensome and diverse utilization management policies for opioid alternatives likely results in slower adoption and implementation of these treatments. By contrast, coordination of pharmacologic and nonpharmacologic treatment options, such as through step therapy requirements for opioids, could incentivize the use of treatments with better evidence and with less addictive potential. Our observation that only 1 plan fully integrated nonpharmacologic therapies into its step therapy requirements for opioid initiation underscores the lack of a comprehensive, evidence-based approach to the specific act of opioid prescribing, as well as the broader issue of treating chronic pain.^9^ The lack of alignment of pharmacy and medical policies within individual payers also reinforces the disconnect in linking patients to the right care at the right time in the course of their disease. While physician overprescribing of opioids may have fueled the opioid epidemic, inconsistencies between payer policies and evidence-based practices for opioid prescribing pose significant barriers to ensuring that patients with chronic pain receive high-quality, multimodal, evidence-based treatment.

The quantity limits used by the plans we examined were largely based on a 30-day supply or longer, with no difference based on dose or initial vs subsequent prescription. These types of limits may have limited utility for reducing nonmedical use, given recent research showing that the probability of long-term opioid use increases sharply among patients receiving just 5 or more days of opioid therapy at the time of initial prescription.^28^ Recent legislation in a number of states have set 7-day or shorter limits on initial opioid prescriptions, and CVS/Caremark, one of the nation’s largest pharmacy benefit managers, announced in September 2017 their intention to institute similar limits on initial prescriptions.^21,22,23^ Although more studies are needed to understand the intended and unintended impact of these types of policies, quantity limits represent one of many policies that insurers should consider.

### Limitations

Our study had limitations. First, publicly available documents were not consistently available for all payers, and we have no information on the reliability of the documents we obtained. However, these documents are used by many parties, are regularly updated, and serve as a principal method of communication regarding coverage policies. Second, our analysis focused on a sample of 50 plans and does not include some health systems and payers such as the Veterans Health Administration and workers’ compensation plans. Third, the opioid epidemic is a dynamic and evolving public health crisis and our work suggests that payers continue to modify their policies to respond to a changing marketplace as well as public and population health priorities. This fact underscores the importance of ongoing evaluation of coverage policies over time, including their impact on utilization, processes, and outcomes of care. Fourth, as with all qualitative work, ours may have been subject to our own biases and preconceptions, although we used several approaches to minimize the degree to which this may have influenced our results. We did not explore the rationale behind the implementation of different utilization management tools, such as prescribing limits vs prior authorization. Fifth, we limited our analysis to pharmacologic treatments, yet nonpharmacologic therapies, ranging from physical therapy to acupuncture to counseling, represent important modalities in chronic pain management. Plan coverage, availability, and variation in nonpharmacologic therapies for pain management are not well understood and warrant further study.

## Conclusions

The opioid epidemic is a complex crisis that requires partnership across multiple sectors to respond with effective clinical and public health strategies.^29^ To our knowledge, our analysis is the largest and most comprehensive examination of recent coverage policies among a diverse sample of Medicaid, Medicare Advantage, and commercial insurers in the United States. Our findings point to opportunities among insurers and pharmacy benefit managers to recalibrate the role of opioids in pain care, expand access to opioid alternatives through coverage and reimbursement policies, and measure the impact of such changes on patient outcomes. Furthermore, such efforts must be implemented in the context of a comprehensive suite of interventions that targets drivers of the epidemic.

## Notes

Supplement. **eAppendix.** Selection of Products and Plans **eTable 1.** Payers and Plans Examined **eTable 2.** List of Pharmaceutical Treatments **eTable 3.** Coverage of Opioids Across Medicaid Plans, Medicare Advantage Plans, and Commercial Plans **eTable 4.** Tiering and Out-of-Pocket Costs for Medicare Advantage and Commercial Plans **eReferences**
