# Supplementary material for: Prescription Drug Coverage for Treatment of Low Back Pain Among US Medicaid, Medicare Advantage, and Commercial Insurers
Source: JAMA Netw Open. 2018 Jun 22;1(2):e180235. doi: 10.1001/jamanetworkopen.2018.0235 (PMC6324424; doi:10.1001/jamanetworkopen.2018.0235)
Supplement: Supplement. — eAppendix. Selection of Products and Plans eTable 1. Payers and Plans Examined eTable 2. List of Pharmaceutical Treatments eTable 3. Coverage of Opioids Across Medicaid Plans, Medicare Advantage Plans, and Commercial Plans eTable 4. Tiering and Out-of-Pocket Costs for Medicare Advantage and Commercial Plans eReferences [file jamanetwopen-1-e180235-s001.pdf]

Supplementary Online Content

Lin DH, Jones CM, Compton WM, et al. Prescription drug coverage for treatment of low back pain among US Medicaid, Medicare Advantage, and commercial insurers. *JAMA Netw Open*. 2018;1(2):e180235. doi:10.1001/jamanetworkopen.2018.0235

eAppendix. Selection of Products and Plans

eTable 1. Payers and Plans Examined

eTable 2. List of Pharmaceutical Treatments

eTable 3. Coverage of Opioids Across Medicaid Plans, Medicare Advantage Plans, and Commercial Plans

eTable 4. Tiering and Out-of-Pocket Costs for Medicare Advantage and Commercial Plans

eReferences

This supplementary material has been provided by the authors to give readers additional information about their work.

© 2018 Lin DH et al. *JAMA Network Open*.

eAppendix. Selection of Products and Plans

Selection of Products

First, we identified six therapeutic classes commonly used for the treatment of low back pain: opioids, non-steroidal anti-inflammatory drugs (NSAIDs), antidepressants, anticonvulsants, skeletal muscle relaxants and topical analgesics. Next, we used the Truven Micromedex RED BOOK (2014) and the FDA's Approved Drug Products list to populate these classes with specific products of interest. Lastly, we reviewed this list with clinical experts in the treatment of pain and refined it to reflect both conventional practice and clinical guidelines for low back pain, by excluding some products that were either used infrequently or had limited evidence of efficacy in this area.

Selection of Medicaid Plans

We selected 15 states based on varying demographics, such as large and small populations, wealth, level of urbanicity, and those with disproportionately high rates of injuries and deaths from prescription and non-prescription opioids, such as Ohio, West Virginia and Maine.[1] Within tertiles of FMAP scores, we selected 4-6 states with varying population sizes and geographic regions, as defined by the United States Census Bureau. As a result, we selected 1 to 2 states from each of the 9 geographic regions. Using data from the Kaiser Family Foundation,[2] we selected Medicaid formularies from the largest Managed Care Organization (MCO) in each state, with the exception of one state's largest MCO, whose publicly available formulary listed analgesics in a unique fashion. We instead used the formulary from the second largest Medicaid MCO in this state.

Selection of Medicare Advantage Plans

Since their inception, the proportion of Medicare beneficiaries enrolled in Medicare Advantage ("Part C") has steadily grown, relative to traditional ("fee-for-service") Medicare.[3] In 2017, Medicare Advantage enrollees numbered 19.0 million and accounted for 33% of all Medicare beneficiaries. To maximize representativeness, we selected the same 15 states as for the Medicaid plans, with the exception of Vermont, where we substituted Connecticut, due to Medicare enrollment data availability. Connecticut also has a relatively small population and is located in the same geographic region as Vermont. Our overall selection of states for Medicare Advantage also varied greatly in the number of their Medicare Advantage beneficiaries.

We referred to enrollment data from Medicare.gov to select Medicare Advantage Plan Types, such as health maintenance organization (HMO) or preferred provider organization (PPO) plans, with the largest or second largest enrollment in each state. Meanwhile, we ensured selection of plans from the largest 5 Medicare Advantage insurers, including Aetna, Anthem, Humana, Kaiser Foundation and United Healthcare, as well as a variety of smaller payers. We then selected plans with a variety of star ratings, which are offered for reference by medicare.gov. Star ratings are an evaluation of a plan's overall quality and performance, determined by the Centers for Medicare and Medicaid Services, while taking member experience into consideration.

Selection of Commercial Plans

© 2018 Lin DH et al. *JAMA Network Open*.

We selected 20 plans derived from a total of 7 states, in order to examine multiple plans within the same state. These 7 states were of different population sizes and geographic regions, selected from the list of states for the Medicaid plans. These states also varied in the magnitude by which they were affected by the opioid epidemic. We examined three commercial plans from each of 6 states, and two commercial plans from the last state.

Recent estimates suggest 74.9 million Americans are covered under private insurance.[4] The majority (57%) of these are covered under what's considered large group plans (greater than 51 employees). The remaining individuals are covered under either small group plans (19.6%) or the individual markets (23.3%). We focus our analysis on the small and large group markets, given that these markets have been the predominant provider of private health insurance. To identify plans of interest, we used the Kaiser Family Foundation data on individual states and the largest enrollments in both the large and small group health insurance markets.[5] Of note, there is significant overlap of insurance carriers between the individual, small, and large group markets. For example, the top three carriers in California are the same across all three markets with slight differences in ordering. Additionally, within the same insurance carrier, formularies do not vary much outside of cost-sharing levels, such that a UnitedHealthcare formulary in one state will be similar, if not identical, to the UnitedHealthcare formulary in another state. Once a potential insurance plan was identified, we examined the insurance carrier website for access to the specific state-level formulary. Some plans restricted the plan-specific documents and level of information made publicly available; in these cases, we selected a different plan with large enrollment, but in the same state.

© 2018 Lin DH et al. *JAMA Network Open*.

eTable 1. Payers and Plans Examined

State	Medicaid	Medicare Advantage	Commercial Plan
Arkansas	Arkansas Medicaid	United Healthcare	---
California	MediCal	Kaiser Foundation	Anthem Blue Cross Blue Shield
Colorado	Health First Colorado	Kaiser Foundation	---
Connecticut	---	Aetna	---
Florida	Florida Medicaid	Humana	---
Georgia	Georgia Medicaid	Humana	---
Idaho	Idaho Medicaid	Anthem	---
Maine	MaineCare	Martin's Point Generations Advantage	Aetna<br>Anthem
Michigan	Michigan Medicaid	Anthem	---
Missouri	MO HealthNet	Aetna	---
New York	New York Medicaid	Healthfirst	Aetna<br>Empire Blue Cross<br>Excellus BCBS
North Dakota	North Dakota Medicaid	Medica	Blue Cross Blue Shield North Dakota<br>Medica
Ohio	Ohio Medicaid	MediGold	Anthem<br>Medical Mutual of Ohio<br>United Healthcare
Texas	Texas Medicaid	United Healthcare	HCSC<br>United Healthcare
Vermont	Vermont Medicaid	---	---
West Virginia	West Virginia Medicaid	Aetna	Aetna<br>Highmark

© 2018 Lin DH et al. *JAMA Network Open*.

eTable 2. List of Pharmaceutical Treatments

Medications	Release Type	Short- or Long-Acting	Schedule	Abuse-Deterrent Formulation?
OPIOIDS				
Buprenorphine	Extended release	Long	III	No
Buprenorphine	Extended release	Long	III	No
Codeine	Immediate release	Short	II	No
Codeine-acetaminophen	Immediate release	Short	II	No
Codeine-carisoprodol-aspirin	Immediate release	Short	II	No
Fentanyl, transdermal	Extended release	Long	II	No
Hydrocodone (extended)	Extended release	Long	II	Yes
Hydrocodone-acetaminophen	Immediate release	Short	II	No
Hydrocodone-ibuprofen	Immediate release	Short	II	No
Hydromorphone	Immediate release	Short	II	No
Hydromorphone (extended)	Extended release	Long	II	No
Meperidine hydrochloride	Immediate release	Short	II	No
Methadone	Immediate release	Long	II	No
Morphine sulfate	Immediate release	Short	II	No
Morphine sulfate (extended)	Extended release	Long	II	No
Morphine-naltrexone (extended)	Extended release	Long	II	Yes
Oxycodone	Immediate release	Short	II	No
Oxycodone (extended)	Extended release	Long	II	No
Oxycodone-acetaminophen	Immediate release	Short	II	No
Oxycodone-aspirin	Immediate release	Short	II	No
Oxycodone-ibuprofen	Immediate release	Short	II	No
Oxycodone-naltrexone (extended)	Extended release	Long	II	Yes

© 2018 Lin DH et al. *JAMA Network Open*.

Oxymorphone	Immediate release	Short	II	No
Oxymorphone (extended)	Extended release	Long	II	No
Pentazocine-naloxone	Immediate release	Short	IV	No
Tapentadol	Immediate release	Short	II	No
Tapentadol (extended)	Extended release	Long	II	No
Tramadol	Immediate release	Short	IV	No
Tramadol (extended)	Extended release	Long	IV	No
Tramadol-acetaminophen	Immediate release	Short	IV	No
TOPICAL ANALGESIC				
Diclofenac Sodium	Immediate release			
Lidocaine	Immediate release			
ANTICONVULSANT				
Gabapentin	Immediate release			
Gabapentin (extended)	Extended release			
Gabapentin enacarbil (extended)	Extended release			
Pregabalin	Immediate release			
ANTIDEPRESSANT				
Amitriptyline	Immediate release			
Desipramine	Immediate release			
Desvenlafaxine (extended)	Extended release			
Duloxetine (delayed)	Delayed release			
Imipramine	Immediate release			
Levomilnacipran (extended)	Extended release			
Milnacipran	Immediate release			
Nortriptyline	Immediate release			
Venlafaxine	Immediate release			
Venlafaxine (extended)	Extended release			

© 2018 Lin DH et al. *JAMA Network Open*.

NSAIDs				
Celecoxib	Immediate release			
Diclofenac (extended)	Extended release			
Diclofenac Potassium	Immediate release			
Diclofenac Sodium (delayed)	Delayed release			
Ibuprofen	Immediate release			
Meloxicam	Immediate release			
Naproxen	Immediate release			
Naproxen (delayed)	Delayed release			
Naproxen (extended)	Extended release			
Piroxicam	Immediate release			
SKELETAL MUSCLE RELAXANT				
Carisoprodol	Immediate release			
Cyclobenzaprine	Immediate release			
Cyclobenzaprine (extended)	Extended release			
Metaxalone	Immediate release			
Methocarbamol	Immediate release			
Tizanidine	Immediate release			

© 2018 Lin DH et al. *JAMA Network Open*.

eTable 3A. Coverage of Opioids Across Medicaid Plans (N=15)

	**Plan Identification Number**														
	**1**	**2**	**3**	**4**	**5**	**6**	**7**	**8**	**9**	**10**	**11**	**12**	**13**	**14**	**15**
Buprenorphine (transdermal)[a]	Yes	No	No	No	Yes	No	Yes	No	Yes	No	Yes	Yes	Yes	Yes	No
Buprenorphine (sublingual)[a]	Yes	No	No	No	Yes	No	No	No	Yes	No	Yes	Yes	Yes	Yes	No
Codeine	Yes	Yes	Yes	Yes	Yes	Yes	Yes	Yes	No	No	No	Yes	No	Yes	Yes
Codeine-acetaminophen	Yes	Yes	Yes	Yes	Yes	Yes	Yes	Yes	No	Yes	Yes	Yes	Yes	Yes	Yes
Codeine-carisoprodol-aspirin	Yes	No	No	No	No	No	Yes	No	No	No	Yes	Yes	Yes	Yes	No
Fentanyl Patch	Yes	Yes	Yes	Yes	No	Yes	Yes	Yes	Yes	Yes	Yes	Yes	Yes	Yes	Yes
Hydrocodone ER	Yes	No	No	No	Yes	No	No	No	Yes	No	Yes	No	Yes	Yes	Yes
Hydrocodone-acetaminophen	Yes	No	Yes	No	Yes	No	Yes	Yes	No	Yes	Yes	Yes	Yes	Yes	Yes
Hydrocodone-ibuprofen	Yes	No	No	No	Yes	No	Yes	No	No	Yes	Yes	No	Yes	Yes	Yes
Hydromorphone	Yes	Yes	Yes	Yes	Yes	Yes	No	Yes	No	Yes	Yes	Yes	Yes	Yes	Yes
Hydromorphone ER	Yes	No	No	No	No	No	Yes	No	Yes	No	Yes	Yes	Yes	Yes	Yes
Meperidine	Yes	Yes	No	No	No	Yes	No	Yes	No	Yes	Yes	Yes	Yes	Yes	Yes
Methadone[a]	Yes	Yes	Yes	Yes	Yes	Yes	Yes	Yes	Yes	Yes	No	Yes	Yes	Yes	Yes
Morphine	Yes	Yes	Yes	Yes	No	Yes	Yes	Yes	No	Yes	Yes	Yes	Yes	Yes	Yes
Morphine ER	Yes	Yes	No	No	Yes	No	Yes	Yes	Yes	Yes	Yes	Yes	Yes	Yes	No
Morphine-naltrexone	Yes	No	No	No	Yes	No	No	No	Yes	No	Yes	Yes	Yes	Yes	Yes
Oxycodone	Yes	Yes	Yes	Yes	Yes	Yes	Yes	Yes	No	Yes	Yes	Yes	Yes	Yes	Yes
Oxycodone ER	Yes	No	No	Yes	Yes	Yes	Yes	Yes	Yes	Yes	Yes	Yes	Yes	Yes	No
Oxycodone-acetaminophen	Yes	No	Yes	No	Yes	No	Yes	No	Yes	Yes	Yes	Yes	Yes	Yes	Yes
Oxycodone-aspirin	Yes	Yes	Yes	Yes	No	No	Yes	Yes	No	Yes	Yes	Yes	Yes	Yes	No
Oxycodone-ibuprofen	Yes	No	No	No	No	No	Yes	No	No	No	Yes	Yes	Yes	Yes	Yes
Oxycodone-naltrexone ER[b]	No	No	No	No	No	No	No	No	No	No	No	No	No	No	No
Oxymorphone	Yes	No	No	No	No	No	No	No	No	No	Yes	Yes	Yes	Yes	Yes

© 2018 Lin DH et al. *JAMA Network Open*.

Oxymorphone ER[b]	Yes	No	No	No	No	No	Yes	Yes	Yes	Yes	Yes	Yes	Yes	Yes	Yes
Pentazocine-naloxone	Yes	No	No	Yes	Yes	Yes	Yes	No	No	No	Yes	Yes	Yes	Yes	Yes
Tapentadol	Yes	No	No	No	Yes	No	Yes	No	No	No	Yes	Yes	Yes	Yes	Yes
Tapentadol ER	Yes	No	No	No	Yes	No	Yes	No	Yes	No	Yes	Yes	Yes	Yes	Yes
Tramadol	Yes	Yes	Yes	Yes	Yes	Yes	Yes	Yes	No	Yes	Yes	Yes	Yes	Yes	Yes
Tramadol ER	Yes	No	No	No	Yes	No	Yes	Yes	Yes	No	Yes	Yes	Yes	Yes	Yes
Tramadol-acetaminophen	Yes	No	No	No	No	No	Yes	Yes	No	Yes	Yes	Yes	Yes	Yes	Yes

Table depicts whether or not an opioid product was on the program’s formulary.
[a]Prescribed for pain; [b] Removed from market during study period.

© 2018 Lin DH et al. *JAMA Network Open*.

eTable 3B. Coverage of Opioids Across Medicare Advantage Plans (N=15)

	Plan Identification Number (Values in cells represent coverage status and tiers)														
	**1**	**2**	**3**	**4**	**5**	**6**	**7**	**8**	**9**	**10**	**11**	**12**	**13**	**14**	**15**
Buprenorphine (transdermal)[a]	NC	4	4	NC	NC	NC	3	NC	4	NC	NC	NC	3	NC	NC
Buprenorphine (sublingual)[a]	NC	4	4	NC	NC	NC	4	NC	NC	NC	NC	NC	4	NC	NC
Codeine	3	2	2	4	3	3	2	NC	2	4	NC	NC	2	3	4
Codeine-acetaminophen	2	2	2	2	3	3	2	2	2	2	2	2	2	2	2
Codeine-carisoprodol-aspirin	NC	2	2	NC	NC	NC	NC	NC	NC	NC	NC	NC	NC	NC	NC
Fentanyl Patch	4	2	2	4	3	4	2	2	2	4	2	4	2	4	4
Hydrocodone ER	NC	4	4	NC	NC	NC	4	NC	NC	NC	NC	NC	4	NC	NC
Hydrocodone-acetaminophen	3	2	2	3	3	3	2	NC	2	3	NC	NC	2	3	3
Hydrocodone-ibuprofen	NC	2	2	3	4	4	2	NC	2	3	NC	NC	2	NC	3
Hydromorphone	2	2	2	3	3	3	2	2	2	3	2	3	2	2	3
Hydromorphone ER	4	2	2	NC	NC	NC	2	NC	NC	NC	NC	NC	2	4	NC
Meperidine	NC	2	2	NC	3	3	NC	NC	NC	NC	NC	NC	NC	NC	NC
Methadone[a]	3	2	2	3	3	3	2	2	2	3	2	3	2	3	3
Morphine	3	3	3	2	3	3	2	2	2	2	2	3	2	3	2
Morphine ER	NC	2	2	4	4	4	2	NC	4	4	NC	NC	2	NC	4
Morphine-naltrexone	3	4	4	NC	NC	3	4	NC	NC	NC	NC	NC	4	3	NC
Oxycodone	2	2	2	3	3	3	2	2	2	3	2	3	2	2	3
Oxycodone ER	NC	2	2	NC	NC	NC	NC	NC	NC	NC	NC	NC	NC	NC	NC
Oxycodone-acetaminophen	3	2	2	3	3	3	2	2	2	3	2	3	2	3	3
Oxycodone-aspirin	3	2	2	3	4	4	2	NC	2	3	NC	NC	2	3	3
Oxycodone-ibuprofen	3	2	2	3	4	4	2	NC	2	3	NC	NC	2	3	3
Oxymorphone ER[b,c]	---	---	---	---	---	---	---	---	---	---	---	---	---	---	---
Oxymorphone	NC	2	2	NC	NC	NC	2	NC	4	NC	NC	NC	2	NC	NC
Oxymorphone ER[c]	NC	2	2	NC	NC	NC	NC	NC	4	NC	NC	NC	NC	NC	NC

© 2018 Lin DH et al. *JAMA Network Open*.

Pentazocine-naloxone	NC	2	2	NC	3	3	NC	NC	NC	NC	NC	NC	NC	NC	NC
Tapentadol	NC	4	4	NC	NC	NC	4	NC	4	NC	NC	NC	4	NC	NC
Tapentadol ER	3	4	4	NC	NC	NC	4	NC	NC	NC	NC	NC	4	3	NC
Tramadol	2	2	2	2	2	2	2	2	2	2	2	2	2	2	2
Tramadol ER	4	2	2	NC	NC	NC	2	NC	2	NC	NC	NC	2	4	NC
Tramadol-acetaminophen	2	2	2	3	3	3	2	2	2	3	2	3	2	2	3

NC=Not Covered.

[a]Prescribed for pain.

[b]Oxycodone-naltrexone ER was not offered as an option on medicare.gov to check formulary coverage.

[c]Removed from market during study period.

© 2018 Lin DH et al. *JAMA Network Open*.

eTable 3C. Coverage of Opioids Across Commercial Plans (N=19)

	Plan Identification Number																		
	**1**	**2**	**3**	**4**	**5**	**6**	**7**	**8**	**9**	**10**	**11**	**12**	**13**	**14**	**15**	**16**	**17**	**18**	**19**
Buprenorphine (transdermal)[a]	NC	3	NC	3	2	3	3	2	3	NC	3	4	NC	3	4	3	2	2	NC
Buprenorphine (sublingual)[a]	NC	NC	NC	3	3	NC	3	3	3	NC	NC	3	NC	3	4	3	3	3	NC
Codeine	2	1	1	1	NC	1	1	1	1	3	1	NC	1	NC	4	1	1	1	2
Codeine-acetaminophen	1	1	1	1	1	NC	1	1	1	1	1	1	1	1	1	1	1	1	1
Codeine-carisoprodol-aspirin	1	1	1	1	1	NC	1	1	NC	NC	NC	NC	NC	NC	2	3	2	1	1
Fentanyl Patch	2	1	2	1	1	2	1	1	1	1	1	NC	1	1	2	1	1	1	2
Hydrocodone ER	NC	NC	NC	NC	2	NC	NC	2	3	NC	2	4	NC	3	NC	NC	2	NC	NC
Hydrocodone-acetaminophen	1	1	1	1	NC	1	1	1	1	1	1	NC	1	NC	2	NC	1	1	1
Hydrocodone-ibuprofen	1	1	1	1	1	1	1	1	1	1	1	1	1	1	NC	NC	1	1	1
Hydromorphone	1	1	1	1	1	1	1	1	1	1	1	1	1	1	1	1	1	1	1
Hydromorphone ER	2	1	NC	1	1	2	1	1	1	1	1	NC	1	NC	2	3	3	1	2
Meperidine	1	1	1	1	1	1	1	1	1	NC	1	NC	NC	NC	2	NC	1	1	1
Methadone[a]	1	1	1	1	1	1	1	1	1	3	1	1	1	1	1	1	1	1	1
Morphine	1	NC	1	1	NC	1	1	1	2	2	1	NC	1	NC	3	1	1	1	1
Morphine ER	2	1	NC	1	1	2	1	1	1	3	1	1	NC	1	2	NC	1	1	2
Morphine-naltrexone	NC	NC	NC	NC	3	1	NC	3	3	3	3	4	3	3	NC	3	3	3	NC

© 2018 Lin DH et al. *JAMA Network Open*.

Oxycodone	2	1	1	1	1	1	1	1	1	1	1	1	1	1	2	1	1	1	2
Oxycodone ER	NC	1	NC	NC	1	NC	NC	1	3	3	NC	NC	NC	NC	4	NC	3	1	NC
Oxycodone-acetaminophen	1	1	1	1	1	1	1	1	1	1	1	1	1	NC	2	1	1	1	1
Oxycodone-aspirin	1	1	1	1	1	1	1	1	1	1	1	NC	1	NC	2	NC	NC	1	1
Oxycodone-ibuprofen	1	1	NC	1	1	1	1	1	NC	3	1	NC	1	NC	2	3	1	1	1
Oxycodone-naltrexone ER[b]	NC	NC	NC	NC	NC	NC	NC	NC	NC	NC	NC	NC	NC	NC	NC	NC	NC	NC	NC
Oxymorphone	2	1	NC	1	1	1	1	1	1	1	1	NC	1	NC	2	3	1	1	2
Oxymorphone ER[b]	2	1	NC	1	1	1	1	1	1	3	NC	NC	1	NC	4	1	1	1	2
Pentazocine-naloxone	1	1	1	1	NC	1	1	1	1	NC	NC	NC	NC	NC	2	3	NC	1	1
Tapentadol	NC	NC	NC	3	2	3	3	3	2	NC	2	NC	3	3	4	3	3	3	NC
Tapentadol ER	NC	3	NC	NC	2	3	NC	3	2	NC	2	NC	3	3	4	3	3	3	NC
Tramadol	1	1	1	1	1	1	1	1	1	1	1	1	1	1	NC	1	1	1	1
Tramadol ER	2	1	1	1	1	1	1	1	1	1	1	2	1	NC	4	3	1	1	1
Tramadol-acetaminophen	1	1	1	1	1	NC	1	1	1	1	NC	1	1	1	1	3	1	1	1

NC=Not Covered.

[a]Prescribed for pain.

[b]Removed from market during study period.

© 2018 Lin DH et al. *JAMA Network Open*.

eTable 4. Tiering and Out-of-Pocket Costs for Medicare Advantage and Commercial Plans

	**Tier Placement, Median N (%)[a]**		**Co-payment in U.S. $ (Tiers 1-4) & Coinsurance % (Tier 5)[b], Median (Interquartile Range)**	
	Opioids	Non-opioids	Retail, 30-day supply	Mail Order, 90-day supply
**Medicare Advantage (N=15 plans)**				
Tier 1: Preferred Generics	0 (0)	3 (14)	4 (2, 10)	9 (6, 30)
Tier 2: Generics	5 (29)	9 (41)	17 (11, 20)	39 (26, 60)
Tier 3: Preferred Branded	6 (53)	3 (15)	47 (45, 47)	129 (91, 141)
Tier 4: Non-preferred Drugs	3 (20)	6 (23)	100 (95, 100)	268 (196, 300)
Tier 5: Specialty Drugs	0 (0)	0 (0)	31 (28, 33)	30 (25, 33)
All tiers combined	17 (100)	22 (100)	- - -	- - -
**Commercial (N=19 plans)**				
Tier 1	18 (74)	20 (81)	10 (9, 10)	23 (18, 26)
Tier 2	1 (7)	1 (4)	25 (20, 33)	61 (50, 80)
Tier 3	3 (13)	3 (13)	49 (44, 53)	123 (116, 140)
Tier 4	0 (0)	0 (0)	78 (65, 89)	218 (195, 244)[c]
All tiers combined	23 (100)	26 (100)	- - -	- - -

[a]Values represent the number of opioids or non-opioids on a given tier in the median plan examined. While Medicare Advantage tiers were consistent across plans, commercial formularies varied in number and definition of tiers.
[b]Commercial analyses based on 8 plans for which copayment data was available
[c]Analysis based on 4 plans for which homogeneous copayment information was available

© 2018 Lin DH et al. *JAMA Network Open*.

eReferences

[1]Rudd RA, Seth P, David F, Scholl L. Increases in Drug and Opioid-Involved Overdose Deaths - United States, 2010-2015. MMWR Morb Mortal Wkly Rep. 2016;65:1445-1452.
[2]Kaiser Family Foundation. Medicaid MCO Enrollment. March 2017. Accessible at: https://www.kff.org/other/state-indicator/medicaid-enrollment-by-mco/. (Accessed December 21, 2017).
[3]Kaiser Family Foundation. Medicare Advantage 2017 Spotlight: Enrollment Market Update. June 2017. Accessible at: https://www.kff.org/medicare/issue-brief/medicare-advantage-2017-spotlight-enrollment-market-update/. (Accessed December 21, 2017).
[4]Houchens PR, Clarkson J, Herbold JS, Fohl H. "2015 Commercial Health Insurance: Overview of Financial Results." Milliman. March 2017. Accessible at: www.milliman.com/insight/2017/2015-commercial-health-insurance-Overview-of-financial-results/. (Accessed on December 20, 2017).
[5]Kaiser Family Foundation. Health Insurance Market Competitiveness. 2014. Accessible at: https://www.kff.org/state-category/health-insurance-managed-care/insurance-market-competitiveness/. (Accessed December 21, 2017).

© 2018 Lin DH et al. *JAMA Network Open*.
